# Processing of Electronic Medical Records for Health Services Research in an Academic Medical Center: Methods and Validation

**DOI:** 10.2196/10933

**Published:** 2018-12-21

**Authors:** Nabilah Rahman, Debby D Wang, Sheryl Hui-Xian Ng, Sravan Ramachandran, Srinath Sridharan, Astrid Khoo, Chuen Seng Tan, Wei-Ping Goh, Xin Quan Tan

**Affiliations:** 1 Centre for Health Services and Policy Research Saw Swee Hock School of Public Health National University of Singapore Singapore Singapore; 2 Regional Health System Planning Office National University Health System Singapore Singapore; 3 Saw Swee Hock School of Public Health National University of Singapore Singapore Singapore; 4 University Medicine Cluster National University Hospital Singapore Singapore

**Keywords:** health services, electronic medical records, data curation, validation studies

## Abstract

**Background:**

Electronic medical records (EMRs) contain a wealth of information that can support data-driven decision making in health care policy design and service planning. Although research using EMRs has become increasingly prevalent, challenges such as coding inconsistency, data validity, and lack of suitable measures in important domains still hinder the progress.

**Objective:**

The objective of this study was to design a structured way to process records in administrative EMR systems for health services research and assess validity in selected areas.

**Methods:**

On the basis of a local hospital EMR system in Singapore, we developed a structured framework for EMR data processing, including standardization and phenotyping of diagnosis codes, construction of cohort with multilevel views, and generation of variables and proxy measures to supplement primary data. Disease complexity was estimated by Charlson Comorbidity Index (CCI) and Polypharmacy Score (PPS), whereas socioeconomic status (SES) was estimated by housing type. Validity of modified diagnosis codes and derived measures were investigated.

**Results:**

Visit-level (N=7,778,761) and patient-level records (n=549,109) were generated. The International Classification of Diseases, Tenth Revision, Australian Modification (ICD-10-AM) codes were standardized to the International Classification of Diseases, Ninth Revision, Clinical Modification (ICD-9-CM) with a mapping rate of 87.1%. In all, 97.4% of the ICD-9-CM codes were phenotyped successfully using Clinical Classification Software by Agency for Healthcare Research and Quality. Diagnosis codes that underwent modification (truncation or zero addition) in standardization and phenotyping procedures had the modification validated by physicians, with validity rates of more than 90%. Disease complexity measures (CCI and PPS) and SES were found to be valid and robust after a correlation analysis and a multivariate regression analysis. CCI and PPS were correlated with each other and positively correlated with health care utilization measures. Larger housing type was associated with lower government subsidies received, suggesting association with higher SES. Profile of constructed cohorts showed differences in disease prevalence, disease complexity, and health care utilization in those aged above 65 years and those aged 65 years or younger.

**Conclusions:**

The framework proposed in this study would be useful for other researchers working with EMR data for health services research. Further analyses would be needed to better understand differences observed in the cohorts.

## Introduction

Secondary use of electronic medical records (EMRs) data by clinicians, researchers, data analysts, and computer scientists has led to promising findings in population health research such as patient-utilization stratification [1], treatment-effectiveness evaluation [2], early detection of diseases [3], and predictive modeling [4]. However, dealing with EMR data is often labor intensive [5] and challenging because of the lack of standardization in data entry, changes in coding procedures over time, and the impact of missing information [6,7]. Processing EMR data for analysis is a critical step in health services research requiring significant time and effort.

Different research teams have described EMR data processing methods [6,8-18]. However, most have focused only on partial aspects of data processing [11-13,15-18] or processing related to a specific disease [6,11,13]. Designing an efficient and structured way to standardize records, process features, link data, and select cohorts for analysis is urgently needed, given the increasing emphasis on big data and analytics to improve patient care and reduce health care expenditure [5,19].

Although the standardization of diagnosis codes of different nosologies or different versions of the same nosology has been reported previously [20,21], the completeness and validity of such mapping is rarely reported. This lack of transparent sharing of code set definitions, construction process, and validity is a barrier to rapid scaling of health services research [22], given its importance and widespread relevance. With the change in coding procedures over time, standardization is hence necessary for longitudinal analyses and cross-period comparisons.

Measures of patient complexity, disease severity, and socioeconomic status (SES) are not readily available in most datasets [23] but have been shown to be useful in population health [24-27] and disease progression studies [28]. Although some studies have used the Charlson Comorbidity Index (CCI) [26,29-31] and drug burden [32,33] to estimate patient complexity, validity of these measures as an estimate for patient complexity has rarely been established in Asia. In the absence of income data, SES is typically derived from area-based income level from census data [34,35], insurance status [36], or property value [37,38]. However, these proxies require additional data as well, which are often not readily available in health care administrative datasets or EMRs.

This study has attempted to address some of these challenges common to the use of EMR data for health services research by detailing a structured framework for EMR data processing. Furthermore, the study proposed and validated methods for standardization of diagnosis codes and construction of disease phenotypes and also proposed and tested derived measures of disease complexity and SES, which could be applicable to other datasets with similar data fields.

## Methods

### Local Electronic Medical Records System and Architecture

**Figure 1:**
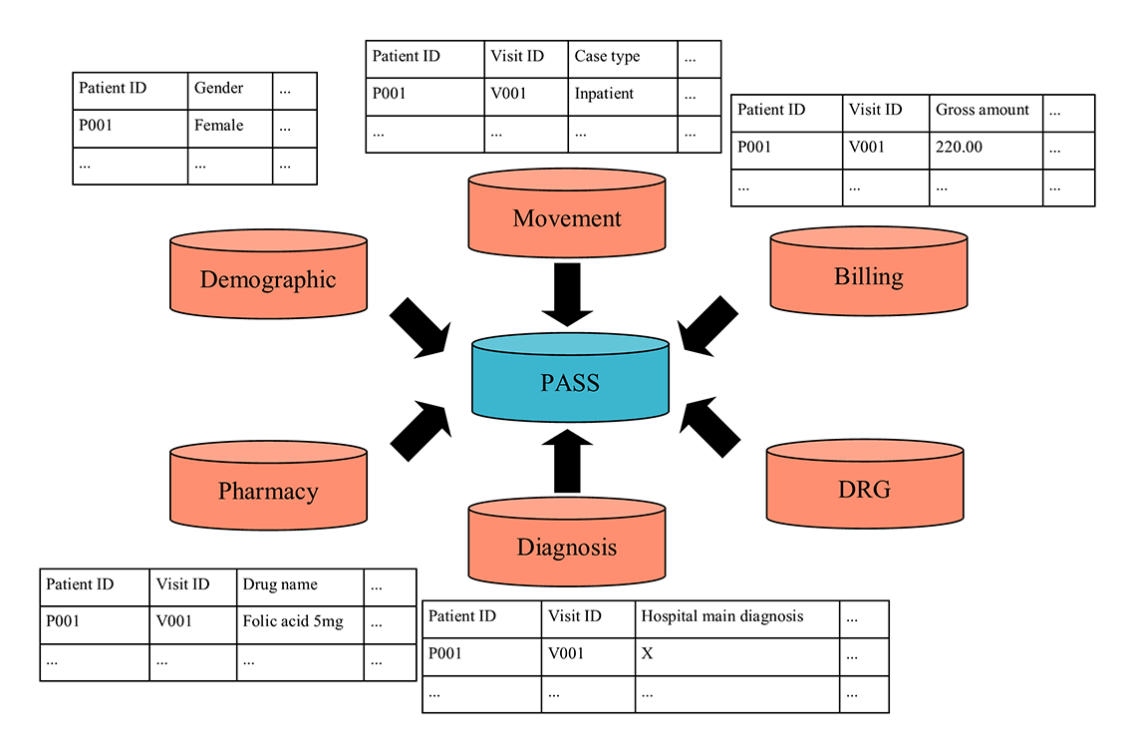
Components of PASS (Patient Affordability Simulation System) database before aggregation, which consist of demographic table (each row is a patient), movement table (each row is a record), billing table (each row is a record or transaction), pharmacy table (each row is a record or transaction), diagnosis table (each row is a record), and diagnosis-related group (DRG) table (not used).

The National University Hospital (NUH) is a 1000-bed Academic Medical Center (AMC) in Singapore [39]. Being 1 of only 2 AMCs in Singapore, its EMR offers an important view of the local patient population, particularly those who have sought care in a tertiary setting. The Patient Affordability Simulation System (PASS) dataset, which this study is premised on, originated from the NUH’s EMR system [40,41]. Specifically, PASS captures information of all patients who visited NUH since 2004, and for this work, we examined data from 2005 to 2013. PASS information is organized in 6 tables: (1) demographic, (2) movement, (3) billing, (4) pharmacy, (5) diagnosis, and (6) diagnosis-related group (DRG) as depicted in Figure 1.

**Figure 2:**
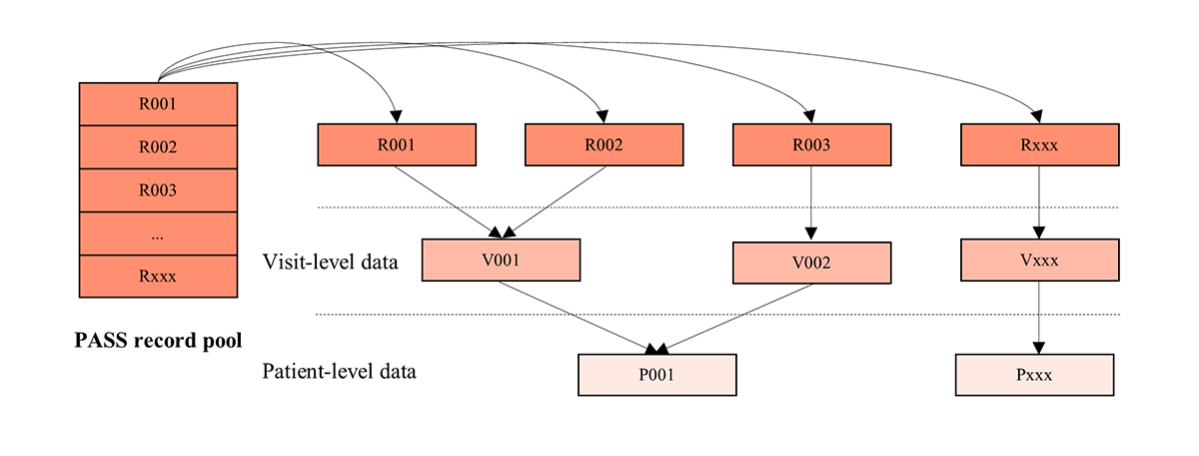
Flow of aggregation from records (before aggregation) to visits and then to patients after aggregations. PASS: Patient Affordability Simulation System.

The cascade architecture of PASS is patient → visit → record as shown in Figure 2 where record is the basic row element for (2) to (5) before aggregation. Five PASS tables were used in our study. The DRG table was not used, as the information captured is a subset of the more comprehensive International Classification of Diseases (ICD) codes found in the diagnosis table.

Patient ID is common in each table and Visit ID is available across (2) to (5). These IDs were used to link features across tables.

### Standardizing and Phenotyping of Diagnosis Codes With Quality Validation

The National University Hospital EMR system adopted International Classification of Diseases, Ninth Revision (ICD-9), Clinical Modification (CM) codes before 2010 and then migrated to the more updated the International Classification of Diseases, Tenth Revision (ICD-10), Australian Modification (AM) codes afterward. To standardize the ICD codes, we transformed ICD-10-AM codes to ICD-9-CM format using Australia Consortium for Classification Development (ACCD) backward mapping tables [42]. ICD-10 is more precise than ICD-9 (ie, there could be multiple ICD-10 codes for each ICD-9, providing greater granularity such as distinguishing the site [left vs right] of pathology). Due to the added granularity, majority of ICD-10 codes cannot be represented by forward mapping of ICD-9 codes [43]. As forward mapping from ICD-9 to ICD-10 and backward mapping from ICD-10 to ICD-9 differ in terms of scope and coverage, both approaches run the risk of ambiguous mappings and loss of information [44,45]. ICD-10 codes also form a significantly smaller portion of diagnosis codes in our database. In this regard, backward mapping of ICD-10 codes to ICD-9 would minimize the impact of above-mentioned risks.

**Figure 3:**
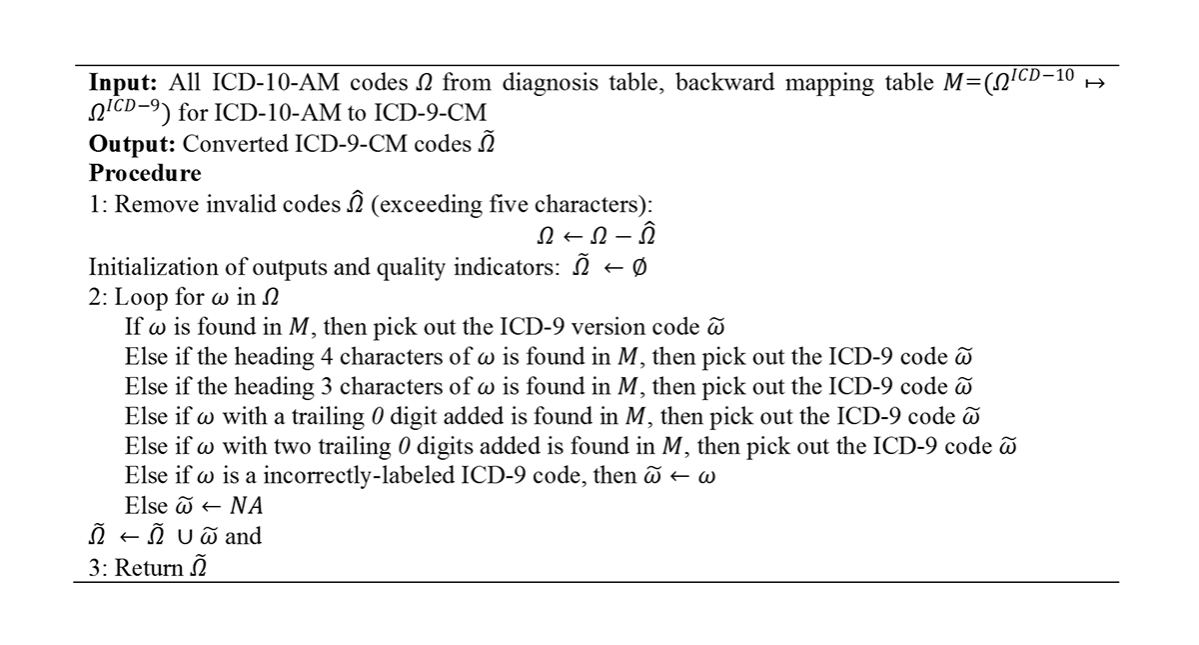
Pseudocode for converting International Classification of Diseases, Tenth Revision, Australian Modification (ICD-10-AM) codes to International Classification of Diseases, Ninth Revision, Clinical Modification (ICD-9-CM) codes.

In transformation to the ICD-9-CM, if an ICD-10-AM code could not be directly mapped to ICD-9-CM using the ACCD backward mapping tables, it will undergo truncation (ie, truncation down to 3 heading digits) or zero addition (ie, addition of up to 2 trailing *0* digits to the ICD code). Mapping will be performed again thereafter. ICD-10-AM codes that were left unmapped after code modification were excluded from further analyses. A diagnosis will be classified as primary diagnosis (PD) if it is indicated as the hospital’s main diagnosis (may be referred as principal diagnosis in other systems [46]). Otherwise, it will be classified as a secondary diagnosis (SD). All PD and SD codes were standardized to ICD-9-CM format. Figure 3 describes the code standardization approach in detail.

As the ACCD backward mapping table is well established and defined, we regard the mapping from original ICD-10-AM codes (no truncations or zero additions) to ICD-9-CM codes as valid [42]. Therefore, to determine the quality of mapping, only those with truncations and zero additions during mapping were examined. We sampled 151 unique ICD-10-AM codes that underwent truncation or zero addition (modified) during the conversion. These 151 codes comprised 23.1% of total 653 unique ICD-10-AM codes that were modified. Thereafter, 2 physicians independently reviewed and rated the validity of the mapping from ICD-10-AM to ICD-9-CM for these sampled codes. List of disagreements in terms of validity of the mapping was generated at the end of the rating exercise and shared between the 2 physicians to reconcile differences through discussions. In the event where disagreement could not be resolved, a third physician would then be brought in. In our study, the 2 physicians managed to reconcile differences without the involvement of the third physician. The ratio of valid mappings after reconciling rating differences by the 2 physicians was then calculated to validate our code standardization approach. Similar method of validating diagnosis codes has been documented in other studies [47-49].

ICD codes have good utility for clinical research where the researcher needs the granularity for identification and attribution of pathology at an individual level [20,50]. However, for health services research, broader classification and coding methods such as the Clinical Classification Software (CCS) by Agency for Healthcare Research and Quality [51] demonstrate utility as there is sufficient granularity at a population level, yet reduced sparsity [52-54].

To extend the utility of our dataset to support health services research, we sought ways to phenotype the more than 10,000 ICD-9-CM codes (both PD and SD) into meaningful groups. To that end, we grouped the ICD-9-CM codes (including those converted from ICD-10-AM codes) using CCS to 283 mutually exclusive disease categories (eg, *essential hypertension* and *cancer of breast*). For ICD codes that could not be classified directly using CCS, the approach outlined in Figure 3 was adopted as well. Validation of the ICD-9-CM codes that underwent truncation or zero addition in the phenotyping was conducted using the same methodology as described above. In total, 361 (20.7%) unique ICD-9-CM codes of the total 1747 unique ICD-9-CM codes that were modified during the phenotyping were sampled for this purpose.

### Cohort Generation and Feature Processing

#### Generating Visit and Patient-Level Records

The PASS EMR had captured data at the record level. For meaningful analysis to be performed, the database had to be processed to generate visit-level and patient-level records. Visit-level records capture information related to a single encounter with NUH. Patient-level records capture information on the patient himself as well as information related to the visits accumulated over the study period.

The 2 types of unique identifiers used for record linkage are Patient ID and Visit ID. To generate visit-level records, we used Visit ID to aggregate records within each table (eg, all bills for a visit) and then fully join the data for each visit by drawing on data across tables (ie, linking the movement, billing, pharmacy, and diagnosis information to provide more complete utilization and clinical details for each visit). Age and date exclusion criteria were applied to 3 of the tables (2-4) before the join. The diagnosis table was then also filtered using Visit ID from the other tables (2-4) to filter out diagnoses not related to visits within our cohort after applying the earlier exclusion criteria. The tables (2-5) were fully joined thereafter. The joined data were further linked to demographics through Patient ID. Patient-level records were then generated by aggregating visits by Patient ID. Patient-related exclusion criteria were then applied after obtaining patient-level records.

Aggregation and analysis were undertaken using *R* version 3.2.0 [55]. *R* package *multidplyr* [56] was used for efficient parallel aggregation.

#### Exclusion Criteria

The following exclusion criteria were applied to streamline the data for subsequent analysis:

- Any inpatient visit with admission date before 2005 or discharge date after 2013 was dropped. This ensured that the entire period of each inpatient visit was captured.
- Visits when patients were aged less than 21 years were excluded in this study as subsequent analysis is focused on adult patients.
- Patients with no PD were excluded.
- Patients with birth years 1900 or earlier were excluded (patients without birth date information were assigned a default 1900 as the birth year; hence, they were excluded from the study).
- Patients with invalid diagnoses (eg, male patients with diagnoses of pregnancies and female infertility) were removed.

The final cohort analyzed was an adult cohort aged 21 years and above, with valid age and at least one PD record.

#### Preparing Primary and Secondary Variables

The main source variables in the database had to be extracted and processed to generate secondary variables useful for cohort profiling and other health services research. In addition, we attempted to generate proxies for clinical and socioeconomic indicators unavailable in the dataset, namely, disease complexity and SES. Summary and details of all the extracted variables can be found in the Multimedia Appendices 1-3.

On the basis of the source variables from the 5 PASS tables, we generated a series of secondary variables falling in categories of (1) demographics (including SES), (2) health care utilization, (3) disease indicators, and (4) disease complexity. For categorical source variables, we created dummy variables for visits, such as whether a visit is an emergency department (ED) visit or whether it has a specific CCS disease, and then we aggregated them to patient-level by adding new categories, summation, or logic operation. For numerical source variables, such as inpatient length-of-stay (LOS), hospital charges, and the components, a simple summation over all visits led to the features at patient-level. Hospital charges (full cost of care before subsidy) was adjusted using Monetary Authority of Singapore Web-based inflation calculator [57] for health goods and services to 2015 levels before the aggregation to patient-level.

#### Estimating Disease Complexity and Validation of Measures

As clinical indicators and investigation results [23] that provide information on disease severity and patient complexity were not available in the dataset, we introduced 2 measures to estimate disease complexity—CCI [58] and Polypharmacy Score (PPS) [59]. CCI and PPS have both been shown to be good measures of patient comorbidity and complexity in many studies [26,29-33].

In our dataset, the Charlson comorbidities were identified using ICD-9-CM codes [58], and both PD and SD codes were considered for each patient. *R* package *icd* [59] was used to calculate CCI [28]. PPS quantifies drug burden, and high drug burden is usually reflective of more severe disease or greater comorbidity [60]. PPS at the visit-level was defined as the number of unique drugs dispensed in a visit, and PPS at patient-level was defined as the maximum PPS value at visit-level for that patient across all visits. When computing the PPS, nonprescription drugs and devices were excluded.

Validity of CCI and PPS were assessed to ensure that these measures were consistent with theoretical understanding and literature. To assess convergent validity, Spearman rank correlation between PPS and CCI was computed. This measures the degree to which PPS and CCI, that should be measuring disease complexity, are in fact related. To assess criterion validity, Spearman rank correlations between health care utilization (number of inpatient, specialist outpatient clinic [SOC] and ED visits) and PPS and CCI were computed. This measures the extent to which higher CCI and PPS is associated with higher health care utilization, under the assumption that clinically complex patients require more health care utilization [61]. The 95% CIs of the correlations were adjusted for multiple comparisons using Holm method. The health care utilization measures were also regressed on CCI and PPS separately, controlling for demographic variables and observed period to further ascertain its criterion validities. Log-linked negative binomial generalized linear models were used to perform the regression analyses. Missing values are removed pair-wise for the regression analyses in this study. Our methods to assess validity of these proxy measures are similar to methods used in numerous other studies [62-64].

#### Estimating Socioeconomic Status and Validation of Measure

To estimate the SES of PASS patients, we used housing type as a proxy, given the lack of a direct indicator of SES in the dataset. We then validated the use of housing type as a proxy for SES as part of the study.

Each residential block and house in Singapore has a postal code assigned. Using the postal code data of each patient, we were able to determine the block and, consequently, housing type for each patient. The latest postal codes captured in PASS EMR were used, as patients’ past addresses were not available. For all Housing Development Board (HDB) blocks (public housing), we obtained information of flat types by postal codes collected using OneMap Singapore [65] from the official HDB website [66]. The full HDB flat type list includes rental flats, studios, 1- to 5-room flats, and other executive flats. We then grouped the flat types by size as follows: rental to 2-room, 3-room, 4-room, and 5-room to executive flats. If a housing block comprised multiple flat types, it was assigned to the flat type with the largest proportion in that block. Residents living in private condominiums or landed properties were classified as private housing and were identified based on a postal code list of private housing provided by a collaborative research team. Blocks with postal codes not belonging to either lists were defined as nonresidential. Patients with postal codes of nonresidential buildings or with no valid postal codes were assigned with a missing value.

Criterion validity of housing type as a proxy for SES was assessed through studying the relationship between housing type and 2 measures: (1) subsidy status and (2) relative subsidy received (RSR). Subsidy status indicates whether a patient received government subsidized care or nonsubsidized (ie, private) care where nonsubsidized care is costlier and involves higher out-of-pocket payments. Typically, the lower the income level of an individual, the more likely one is to opt for subsidized care given the lower cost [67]. RSR indicates the proportion of the cumulative hospital charges that were paid for with government subsidies. In Singapore, the amount of subsidy one is eligible for and receives is dependent on the income level of the individual [68]. The lower the income level, the more subsidies one is eligible for and a higher percentage of bill will be subsidized. Both subsidy status and RSR were used to validate our SES proxy using Pearson chi-square (χ^2^) and Kruskal-Wallis rank-sum test [69,70], respectively, assuming that lower income groups are more likely to opt for subsidized care and that RSR increases with decreasing income. Subsidy status and RSR were also regressed on SES to further ascertain its criterion validity while controlling for nationality (Singaporean vs non-Singaporean). Multinomial logistic and linear regression models were used to perform the regression analyses.

## Results

### Overview of Electronic Medical Records Aggregation in Patient Affordability Simulation System

**Figure 4:**
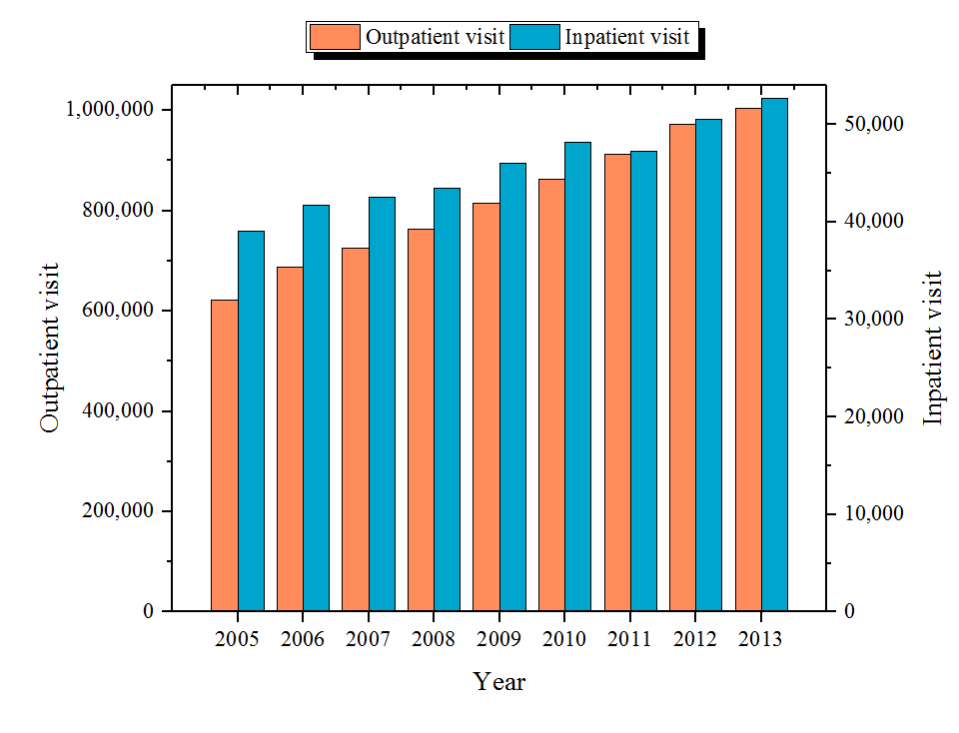
Annual frequency of outpatient and inpatient visits in the cohort.

**Figure 5:**
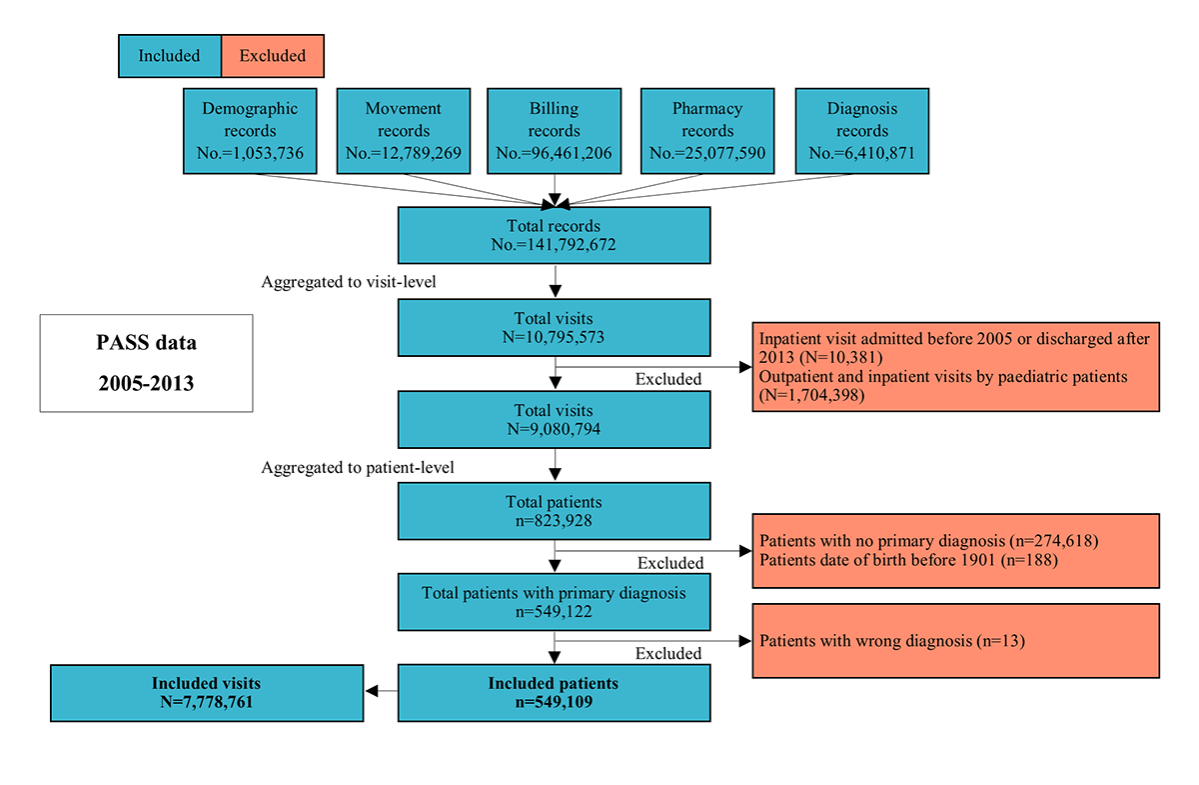
Records, visits, and patients in Patient Affordability Simulation System (PASS) Electronic Medical Records (EMR) aggregation. No.: number of records; N: number of visits; n: number of patients.

Among 10,795,573 visits during the study period, 7,778,761 satisfied our inclusion criteria and constitute our visit-level data. The visit-level data comprised 7,367,495 outpatient visits and 411,266 inpatient visits. An increasing trend was observed in the number of visits from 2005 to 2013 (Figure 4). The visit-level data were subsequently aggregated to the patient-level data, resulting in a cohort of 549,109 adult patients. The flowchart of EMR processing and cohort generation is depicted in Figure 5.

### Mapping Rates and Validation for International Classification of Diseases-10-Australian Modification to International Classification of Diseases-9-Clinical Modification Conversion

There was a total of 4,842,705 diagnoses belonging to our patient cohort after visit-level aggregation, of which 19.2% was coded in ICD-10-AM, with the remainder in ICD-9-CM. The ICD-10-AM codes in our cohort were standardized to ICD-9-CM codes with a mapping rate of 90.3% for PD codes, 78.2% for SD codes, and 81.4% overall using ACCD backward mapping tables. This resulted in 4,670,111 ICD-9-CM codes in the cohort, with 16.2% converted directly from ICD-10-AM by the ACCD backward mapping table. As mentioned in the Methods section, the ACCD backward mapping table has been validated previously; hence, the team regarded these 16.2% of codes that were mapped directly through ACCD as valid. Detailed statistics for code mapping rates are presented in Table 1.

In addition, there were 172,594 codes that could not be mapped through ACCD. Of these, 23,800 (13.79%) ICD-10-AM codes were converted after truncation and 29,005 (16.80%) converted after zero addition (Table 2). These 52,805 ICD-10-AM codes that underwent code modification translated to 653 unique ICD-10-AM codes or 8.9% of the 7373 unique codes that were converted in total. The 52,805 codes accounted for only 6.5% of the 810,459 ICD-10-AM codes that were converted. Validation on a sample of the 653 codes that underwent code modification as part of mapping process was performed. Out of the 151 sampled unique codes, 137 (90.7%) were rated to have valid mappings by the physicians (Table 3).

In total 810,459 (87.1%) of the total ICD-10-AM codes were successfully converted to ICD-9-CM codes (97.2% of PD and 83.5% of SD). These converted codes and the original ICD-9-CM codes form a pool of 4,722,916 (4,722,916/ 4,842,705, 97.5%) ICD-9-CM codes in our cohort. The unmapped codes, which consisted of 119,789 (12.9%) of the total ICD-10-AM codes, or 471 unique codes were excluded.

**Table 1:** International Classification of Diseases (ICD) and Clinical Classification Software (CCS) codes mapping rates.

Diagnosis		Primary	Secondary	Primary and secondary
**Total diagnosis codes^a^, n** **(% of total codes)**				
	Total	1,718,049 (100.00)	3,124,656 (100.00)	4,842,705 (100.00)
	ICD^b^-9-CM^c^	1,470,473 (85.59)	2,441,984 (78.15)	3,912,457 (80.79)
	ICD-10-AM^d^	247,576 (14.41)	682,672 (21.85)	930,248 (19.21)
Total ICD-9-CM codes after ACCD^e^ backward mapping*,* n (%)		1,693,940 (98.60)	2,976,171 (95.25)	4,670,111 (96.44)
Total ICD-9-CM codes after ACCD backward mapping and code modification*,* n (%)		1,711,180 (99.60)	3,011,736 (96.39)	4,722,916 (97.53)
Total CCS^f^ codes after phenotyping, n (% of total ICD-9-CM codes after conversion)		1,402,931 (81.99)	2,775,931 (92.17)	4,178,862 (88.48)
Total CCS codes after phenotyping and code modification, n (% of total ICD-9-CM codes after conversion)		1,696,963 (99.17)	2,901,525 (96.34)	4,598,488 (97.37)

^a^Total number of diagnosis codes from cohort (nonunique codes).

^b^ICD: International Classification of Diseases.

^c^CM: clinical modification.

^d^AM: Australian modification.

^e^ACCD: Australian Consortium for Classification Development.

^f^CCS: Clinical Classification Software.

**Table 2:** Proportion of International Classification of Diseases (ICD) codes that underwent truncation or zero addition.

Diagnosis	No modification, n (% of total mapped)	Modified		Total mapped, n (% of total codes)	Total codes, n (% of total codes)
		Truncated, n (% of total mapped)	Zero added, n (% of total mapped)		
ICD^a^-10-AM^b^ codes converted to ICD-9-CM^c^ (unique codes)	6720 (91.14)	195 (2.64)	458 (6.21)	7373 (94.00)	7844 (100.00)
ICD-10-AM codes converted to ICD-9-CM	757,654 (93.48)	23,800 (2.94)	29,005 (3.58)	810,459 (87.12)	930,248 (100.00)
ICD-9-CM^d^ converted to CCS^e^ codes (unique codes)	9220 (84.07)	246 (2.24)	1501 (13.69)	10,967 (88.26)	12,426 (100.00)
ICD-9-CM^d^ converted to CCS Codes	4,178,862 (90.87)	27,240 (0.59)	392,386 (8.53)	4,598,488 (97.37)	4,722,916 (100.00)

^a^ICD: International Classification of Diseases.

^b^AM: Australian modification.

^c^CM: clinical modification.

^d^After conversion from ICD-10-AM to ICD-9-CM using Australian Consortium for Classification Development (ACCD) backward mapping tables and code modification.

^e^CCS: Clinical Classification Software.

**Table 3:** Validity rate of International Classification of Diseases (ICD) codes, which underwent truncation or zero addition during standardization and phenotyping.

Diagnosis	Valid, n (%)	Invalid, n (%)	Total sample, n (%)
ICD-9-CM^a^ codes from modified ICD-10-AM^b^ codes	137 (90.7)	14 (9.3)	151 (100.0)
CCS^c^ codes from modified ICD-9-CM	332 (92.0)	29 (8.0)	361 (100.0)

^a^ICD-9-CM: International Classification of Diseases, Ninth Revision, Clinical Modification.

^b^ICD-10-AM: International Classification of Diseases, Tenth Revision, Australian Modification.

^c^CCS: Clinical Classification Software.

### Mapping Rates and Validation for Phenotyping of International Classification of Diseases-9-Clinical Modification Codes

The ICD-9-CM codes were then phenotyped to CCS codes, which resulted in 282 mutually exclusive groups. Out of the 4,722,916 ICD-9-CM codes, 4,178,862 (4,178,862/4,722,916, 88.48%) were converted to CCS codes directly through the CCS. These 4,178,862 (88.48%) are regarded to be valid conversions, given the previous validation done on the CCS [71]. Detailed statistics for code-mapping rates are presented in Table 1.

In addition, 27,240 (27,240/4,722,916, 0.58%) ICD-9-CM codes were converted after truncation and 392,386 (392,386/4,722,916, 8.31%) converted after zero addition (Table 2) through our proposed methodology. These 419,626 ICD-9-CM codes that underwent code modification translated to 1747 unique codes or 15.9% of 10,967 unique codes that were collapsed to CCS codes. Moreover, 332 (332/361, 92.0%) of the 361 sampled unique codes were rated as valid mappings by the physicians (Table 3).

In total, 4,598,488 (97.4%) of the ICD-9-CM codes in our cohort were successfully converted to CCS codes (99.2% of PD and 96.3% of SD; Table 1). The 419,626 codes that underwent code modification accounted for only 9.1% of the 4,598,488 ICD-9-CM codes that were collapsed. The unmapped codes, which consisted of 124,428 (124,428/4,722,916, 2.63%) of the total valid ICD-9-CM codes, or 1459 unique codes were excluded.

### Validation of Proxy Measures

CCI was found to be positively correlated with health care utilization measures, including number of inpatient visits (*ρ*=.54; CI 0.54-0.54; *P*<.001), number of SOC visits (*ρ*=.30; CI 0.29-0.30; *P*<.001), and number of ED visits (*ρ*=.21; CI 0.21-0.21; *P*<.001); PPS was found to have an even stronger correlation with health care utilization measures, with exception being number of ED visits: number of inpatient visits (*ρ*=.74; CI 0.74-0.74; *P*<.001), number of SOC visits (*ρ*=.53; CI 0.53-0.54; *P*<.001), and number of ED visits (*ρ*=.19; CI 0.19-0.19; *P*<.001). CCI and PPS were also found to be positively correlated (*ρ*=.47; CI 0.46-0.47; *P*<.001). On the basis of multivariate regression analysis, which adjusted for gender, race, age, and observed period, health care utilization was expected to increase when there was a unit increase in CCI (Table 4). Number of inpatient visits, SOC visits, and ED visits were expected to change by a factor of 1.46 (*P*<.001), 1.32 (*P*<.001), and 1.23 (*P*<.001), respectively. Health care utilization was also expected to increase when there was a unit increase in PPS; the number of inpatient visits, SOC visits, and ED visits were expected to change by a factor of 1.10 (*P*<.001), 1.08 (*P*<.001), and 1.03 (*P*<.001), respectively.

**Figure 6:**
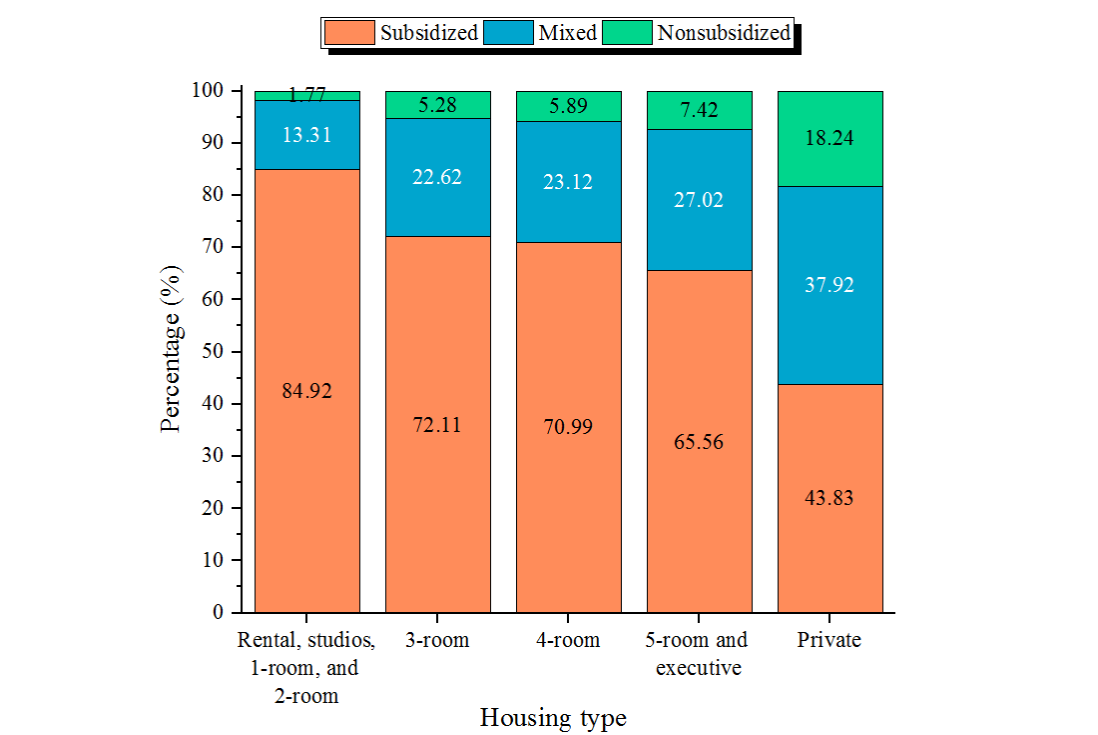
Proportion of subsidy status categories within each housing type.

**Figure 7:**
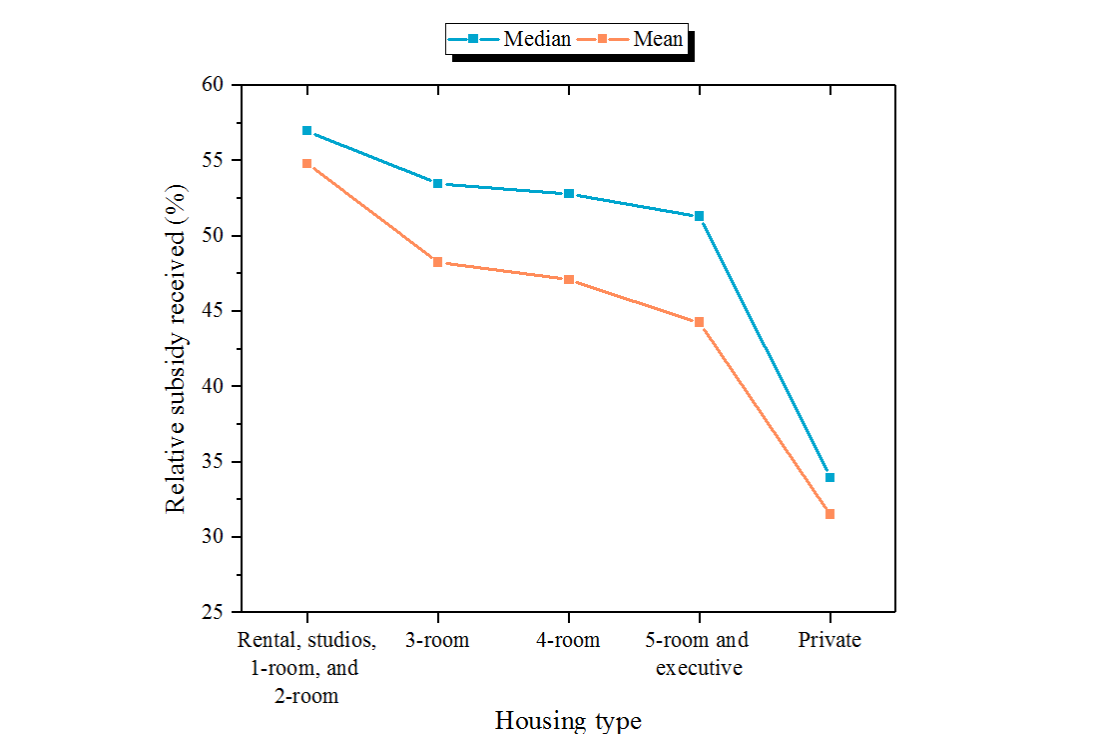
Mean and median relative subsidy received (RSR) within each housing type.

For all the patients with valid housing type data, the proportions by subsidy status categories within each housing type are presented in Figure 6. As housing size decreased, an increase in proportion of subsidized patients was observed—only 43.8% of patients staying in private housing were subsidized compared with 84.9% of patients staying in 2-room or smaller HDB flats. The Pearson chi-square test showed that subsidy status was not independent of housing type (χ^2^_8_=23602, *P*<.001), further confirming the observation. The median and mean RSR of patients by housing type were plotted in Figure 7. Patients who lived in larger housing types tended to have a lower percentage of their bill subsidized (eg, those in 2-room or smaller HDB flats had a median RSR of 57.0% compared with those in private housing with a median RSR of 33.9%). Statistically significant differences in median RSR were observed using Kruskal-Wallis rank-sum test (χ^2^_4_=245232, *P*<.001). The mixed group is a composite group and, hence, it was difficult to interpret the results for this group. On the basis of multivariate regression analysis, which adjusted for nationality, the odds of receiving subsidized care only rather than nonsubsidized care only were higher in patients who lived in smaller housing types when compared with patients who lived in private housing (Table 4). Patients who stayed in 2-room or smaller flats had the highest odds ratio (OR) of 14.43 (*P*<.001), and those who stayed in 5-room flats or executive housing had the lowest OR of 2.97 (*P*<.001). A relatively smaller effect size, but the same trend, was observed when mixed group was compared with nonsubsidized group. Patients who stayed in 2-room flats or smaller and 5-room flats or executive housing had the highest and lowest ORs of receiving both subsidized and nonsubsidized (mixed) care rather than only nonsubsidized care, respectively. The ORs were 3.29 (*P*<.001) and 1.65 (*P*<.001), respectively. RSR was also expected to be higher for patients who stayed in smaller housing after adjusting for nationality (Table 4). Patients who stayed in 2-room or smaller flats were expected to receive 19.0% (*P*<.001) more relative subsidy than those who stayed in private housing, and patients who stayed in 5-room flats or executive housing were expected to receive 9.8% (*P*<.001) more relative subsidy than those who stayed in private housing.

### Profile of Cohort

The detailed demographic, medical, and utilization characteristics of the cohort are shown in Table 5. Overall, most of the 549,109 patients were male, Chinese, aged 30 to 39 years, and lived in a 4-room HDB flat. Of the total patient cohort, 62.0% received only subsidized care in NUH. The total inflated-adjusted hospital charges incurred by the cohort during the 9 years were more than SG $5 billion.

The patients older than 65 years had a greater prevalence of chronic diseases and disease complexity scores as compared with those younger than or at 65 years. They also had almost 7 times the median hospital charges, median LOS that was 3 days longer, and 3 times the median SOC visits during the study period compared with those younger than or at 65 years.

**Table 4:** Multivariate log-linked negative binomial regression on health care utilization, multinomial logistic regression on subsidy status, and linear regression on relative subsidy received (RSR).

Multivariate regression model^a^			Effect (95% CI)	*P* value
**Number of inpatient visits between 2005-2013^b^**				
	CCI^c^		1.47 (1.46-1.47)	<.001
	PPS^d^		1.10 (1.10-1.10)	<.001
**Number of SOC^e^ visits between 2005-2013^b^**				
	CCI		1.32 (1.31-1.32)	<.001
	PPS		1.08 (1.08-1.08)	<.001
**Number of ED^f^ visits between 2005-2013^b^**				
	CCI		1.23 (1.23-1.24)	<.001
	PPS		1.03 (1.03-1.03)	<.001
**Subsidy status between 2005-2013^g^**				
	**Rental, studios, 1-room, and 2-room vs private**			
		Subsidized vs nonsubsidized	14.43 (12.73-16.36)	<.001
		Mixed vs nonsubsidized	3.29 (2.89-3.76)	<.001
	**3-room vs private**			
		Subsidized vs nonsubsidized	4.98 (4.81-5.17)	<.001
		Mixed vs nonsubsidized	1.99 (1.92-2.07)	<.001
	**4-room vs private**			
		Subsidized vs nonsubsidized	4.14 (4.01-4.27)	<.001
		Mixed vs nonsubsidized	1.79 (1.74-1.85)	<.001
	**5-room and executive vs private**			
		Subsidized vs nonsubsidized	2.97 (2.88-3.07)	<.001
		Mixed vs nonsubsidized	1.65 (1.60-1.71)	<.001
**Relative subsidy received between 2005-2013^h^**				
	Rental, studios, 1-room, and 2-room vs private		18.95 (18.57-19.33)	<.001
	3-room vs private		14.43 (14.21-14.64)	<.001
	4-room vs private		12.72 (12.52-12.92)	<.001
	5-room and executive vs private		9.79 (9.59-10.00)	<.001

^a^Eight different models in total.

^b^Effects are exp(β), which can also be interpreted as multiplicative effect.

^c^CCI: Charlson Comorbidity Index.

^d^PPS: Polypharmacy Score.

^e^SOC: specialist outpatient clinic.

^f^ED: emergency department.

^g^Effects are exp(β), which can also be interpreted as odds ratio.

^h^Effects are β.

**Table 5:** Characteristics of patient cohort.

Variables		Patient cohort		
		Total (n=549,109)	21 to 65 years (n=458,069)	>65 years (n=91,040)
Male, n (%)^a^		311,650 (56.76)	267,061 (58.30)	44,589 (48.98)
**Age as at 2013 in years^b^, n (%)^a^**				
	21-29	96,856 (17.64)	96,856 (21.14)	—^c^
	30-39	132,758 (24.18)	132,758 (28.98)	—
	40-49	98,124 (17.87)	98,124 (21.42)	—
	50-59	85,438 (15.56)	85,438 (18.65)	—
	60-69	67,013 (12.20)	44,893 (9.80)	22,120 (24.30)
	70-79	42,327 (7.71)	—	42,327 (46.49)
	≥80	26,593 (4.84)	—	26,593 (29.21)
**Race, n (%)^a^**				
	Chinese	329,544 (60.01)	260,656 (56.90)	68,888 (75.67)
	Indian	70,151 (12.78)	64,483 (14.08)	5,668 (6.23)
	Malay	63,660 (11.59)	54,670 (11.93)	8,990 (9.87)
	Others	85,754 (15.62)	78,261 (17.08)	7,493 (8.23)
Singaporean, n (%)^a^		357,009 (65.02)	279,729 (61.07)	77,280 (84.89)
**Subsidy status, n (%)^a^**				
	Subsidized	340,384 (61.99)	280,810 (61.30)	59,574 (65.44)
	Mixed	154,895 (28.21)	131,943 (28.80)	22,952 (25.21)
	Nonsubsidized	53,830 (9.80)	45,316 (9.89)	8,514 (9.35)
**Housing types, n (%)^a^**				
	Rental, studios, 1-room, and 2-room	14,618 (2.66)	10,881 (2.38)	3,737 (4.10)
	3-room	92,137 (16.78)	71,619 (15.63)	20,518 (22.54)
	4-room	141,637 (25.79)	116,861 (25.51)	24,776 (27.21)
	5-room and executive	119,845 (21.83)	100,116 (21.86)	19,729 (21.67)
	Private	67,152 (12.23)	54,850 (11.97)	12,302 (13.51)
	Missing	113,720 (20.71)	103,742 (22.65)	9,978 (10.96)
**CCS^d^ chronic conditions (primary and secondary), n (%)^a^**				
	Essential hypertension	67,611 (12.31)	29,705 (6.48)	37,906 (41.64)
	Disorders of lipid metabolism	46,060 (8.39)	21,860 (4.77)	24,200 (26.58)
	Diabetes mellitus	43,267 (7.88)	20,725 (4.52)	22,542 (24.76)
	Acute cerebrovascular disease	17,731 (3.23)	7344 (1.60)	10,387 (11.41)
	Asthma	10,177 (1.85)	7588 (1.66)	2589 (2.84)
	Chronic obstructive pulmonary disease and bronchiectasis	8195 (1.49)	3342 (0.73)	4853 (5.33)
**Numerical–total (median; interquartile range)**				
	Inflation-adjusted hospital charges (SG $)	5,177,231,809 (1846; 419-7696)	3,203,726,321 (1363; 352-5366)	1,973,505,488 (9186; 2595-26,100)
	Inpatient visits	411,266 (0; 0-1)	251,891 (0; 0-1)	159,375 (1; 0-2)
	Length-of-stay (days)	2,470,759 (0; 0-3)	1,330,125 (0; 0-2)	1,140,634 (3; 0-14)
	Outpatient visits	7,367,495 (4; 1-13)	5,136,750 (4; 1-11)	2,230,745 (10; 3-29)
	Specialist outpatient clinic visits	4,122,156 (2; 0-8)	2,821,298 (2; 0-6)	1,300,858 (6; 1-18)
	Emergency department visits	834,192 (1; 1-2)	644,230 (1; 1-2)	189,962 (1; 1-2)
	CCI^e^	−(0; 0-0)	−(0; 0-0)	−(1;0-2)
	PPS^f^	−(3; 1-9)	−(3; 1-7)	−(9; 3-17)

^a^As a percentage of respective patient cohorts.

^b^Age of death if patient died before 2013.

^c^Not applicable.

^d^CCS: Clinical Classification Software.

^e^CCI: Charlson Comorbidity Index.

^f^PPS: Polypharmacy Score.

## Discussion

### Principal Findings and Generalizability

Conversion to ICD-10 codes from other codes such as ICD-9 or International Classification of Primary Care is commonly applied in medical or health care studies to increase granularity for identification and attribution of pathology at an individual level. However, given that our dataset is prepped for future health services studies, our primary objective in code standardization was to balance code sparsity with granularity. In this regard, backward mapping from ICD-10 to ICD-9 codes was a more suitable method for this system, and the phenotyping of these standardized codes using broader CCS codes provided different levels of granularity in line with our objectives. Our study showed that standardization of diagnosis codes to ICD-9-CM codes from ICD-10-AM and phenotyping to broader CCS groups through open source-mapping tables could achieve high mapping rates of more than 81% and 88%, respectively. The mapping rates could be further improved through code modification to rates in excess of 97% for ICD PD. Code modification through truncation or zero addition as applied in our study was a robust way of improving the mapping rates as shown by high validity when assessed by independent physicians. Overall, we also showed that bias resulting from code modification was small in our dataset, given that modified codes only constituted less than 2% of total ICD codes and 9% of total CCS codes and that high validity was observed even with these modifications. Given the frequent shifts in ICD codes, these results assure health services researchers that the use of open source-mapping tables together with code modification can rapidly standardize diagnosis coding with low biases and high validity to facilitate retrospective longitudinal analyses. However, we advise caution to researchers who wish to use the ICD-9-CM codes (original and mapped together) directly, without collapsing to CCS codes for their studies as there were ICD-9-CM codes that were unmapped to. Further details on this can be found in Multimedia Appendix 4.

CCI and PPS were introduced as proxy measures of disease complexity. CCI and PPS demonstrated positive correlation with health care utilization measures in keeping with theoretical understanding that patients with more complex disease consume more health care [72]. CCI and PPS were also moderately correlated, which is expected given that both are measures of disease complexity. These associations held true after multivariate regression analysis, demonstrating criterion validity of the measures as proxies for disease complexity. Although other studies found a similar association and effect size between CCI and LOS [73] and between CCI and PPS [74], our study was the first to find such an association between CCI and PPS with health care utilization measures such as inpatient admissions, SOC, and ED visits. These findings support the use of CCI and PPS as measures to stratify patients by complexity and possibly as an aggregate measure of health care utilization, given their correlation with all health care utilization metrics. This finding could be useful in works on profiling, risk stratification, and predictive modeling.

SES is a key determinant of health outcomes and health care utilization [75]. Neither direct measures through individual or household income nor alternate measures of SES such as area-based income were available in our dataset. Hence, we proposed an alternative method of estimating SES using housing type and size because of data availability and the housing landscape in Singapore. In Singapore, the proportion of bill that is subsidized is determined after a rigorous financial assessment and pegged to the income level of the patient (with lower income patients receiving greater levels of subsidy); hence, we hypothesize that lower SES groups would have a greater proportion of their bills subsidized. Given that subsidized care is lower in cost compared with nonsubsidized care, we also expect lower SES groups to opt for subsidized care. In our study, we showed that with decreasing size of housing, the proportion of the hospital bill subsidized increased and the proportion of patients who opted for subsidized care increased. This observation is consistent with our hypothesis that patients who stay in smaller housing types had a greater proportion of their bills subsidized and tended to opt for subsidized care. We have thus shown that in Singapore, housing type and size derived through postal code data are good proxy for income level and SES. Although other studies have shown that staying in rental housing is associated with an increased risk of frequent admissions [76] and readmission [75], as far as the authors are aware, there have not been studies in the Singapore context that have demonstrated the use of housing type as a proxy for SES. Although a missing rate of 20.7% was observed for the housing type variable, this was attributed to foreign patients who registered nonresidential or overseas addresses (86.6% of those with missing housing data are nonresidents). Hence, the missing data are unlikely to bias the findings described above. We were also not able to account for any changes in housing type during the study as the EMR only captured the last postal code of the patient. Public resale data from HDB showed that only 1.6% of public housing units had a change in ownership in 2013 [77]. Hence, we believe it is reasonable to assume that the housing information is static.

Our finding on the suitability of housing type as a proxy for SES is useful, given that most clinical and administrative databases collect addresses but not direct SES information or other proxies. Our method of estimating SES would serve well in countries where methods of estimating SES such as area-based estimates [34,35], insurance status [36], and property value [37,38] are not suitable because of contextual reasons and unavailability of data. For example, area-based estimates may not be suitable in countries where spatial segregation level is low such as in many densely built cities in Asia. In such densely built cities, area-based estimations in effect would need to go down to blocks, which would be similar to using postal codes or addresses. Insurance status is better applied to countries that have high health insurance coverage, which is not the case in most of Asia. Finally, in countries where the real estate market is volatile, property value may be difficult to interpret as a proxy of SES, as the measure would reflect supply and demand dynamics at the point of estimate and numerous extrinsic factors unrelated to SES.

Unlike in countries where zip codes are area-based, the postal codes in Singapore are assigned to each individual building; hence, they serve almost like an address. Housing in Singapore can be divided into 3 main classes: private housing, public housing, and public rental housing. The private housing caters mainly to the upper-middle to upper income groups, whereas the public housing caters to the middle-class population, with 80% of the permanent population living in public housing as owner-occupiers [78]. Eligibility for public housing schemes and new units of certain public housing types depends on household incomes. Moreover, 6% of the public housing stocks are rental units, which serve as social housing for the underprivileged (households with income not exceeding SG $1500) [79]. The housing estates in Singapore were carefully designed to prevent the formation of social enclaves. In the absence of social enclaves (where there is a high concentration of either low or high value housing in an area) [80], area-based estimates are likely to be less valid. With more countries and cities adopting public housing policies and town planning measures to reduce the formation of urban ghettos and sharp sociospatial divisions [81,82] and higher proportions of the population living in tiered public housing [83,84], we do see the applicability of our proposed approach outside of Singapore. Hong Kong is an example of a city with similar ecology where the proposed approach to estimate SES could be used. Although the details may vary, the principle of stratification by type of housing tenure (eg, rental [low-income social housing], public housing, and private housing) first followed by unit size within each tenure type can still be adopted. In countries where urban social residential enclaves exist, 2-stage estimation of SES may be worth exploring by incorporating area-based indices with housing type approach proposed in this study to alleviate the problem of ecological fallacy from solely using area-based indices [85].

Finally, our cohort was found to be similar in profile with the Singapore national population. Comparison with National Census data in 2010 [86] found a similar trend in demographics and housing type, with the exception that our cohort skewed older, which is not unexpected given that health care utilization has been shown to increase with age [87,88]. Patients without PD are excluded from our cohort. These patients exist in our database because it was not mandatory for doctors to key in PD codes for outpatient visits. This would underestimate the number of patients who solely received outpatient care. As such, results from future analysis using the cohort would need to be interpreted with this limitation in mind. Within our cohort, differences in disease profile, disease complexity, and health care utilization could be observed when divided by age.

### Conclusions

With increasing digitization of medical records, use of wearables and Internet-of-Things–connected devices in health care, the amount of data generated by health care systems is growing at a tremendous rate [89,90]. Being able to quickly process and analyze the data generated is key to health care transformation that is needed for sustainability [91]. In this study, we demonstrated how an EMR system in an AMC was processed for health services research. The approach (in whole or part) could be generalized to other EMR systems structured in a similar fashion to support research efforts. In addition, further analyses to better understand differences in the cohorts [1,92] would allow us to better segment the population and eventually predict cost and utilization drivers [4,93]. This is key as we seek to transform care and reduce utilization through targeted interventions and system redesign. The processed database with its multilevel views across time, as well as primary and secondary variables would be integral in achieving these goals.

## Appendix

Multimedia Appendix 1 Basic row elements and column variables in each table in Patient Affordability Simulation System (PASS).

Multimedia Appendix 2 Summary of primary and secondary variables.

Multimedia Appendix 3 Description of extracted primary and secondary variables.

Multimedia Appendix 4 International Classification of Diseases, Ninth Revision, Clinical Modification (ICD-9-CM) codes that were not mapped back from International Classification of Diseases, Tenth Revision, Australian Modification (ICD-10-AM) because of absence of equivalence map in Australian Consortium for Classification Development (ACCD) mapping table.

## Abbreviations

ACCD: Australian Consortium for Classification Development
AM: Australian Modification
AMC: Academic Medical Center
CCI: Charlson Comorbidity Index
CCS: Clinical Classifications Software
CM: Clinical Modification
DRG: Diagnosis-Related Group
ED: emergency department
EMR: electronic medical records
HDB: Housing Development Board
ICD: International Classification of Diseases
ICD-9-CM: International Classification of Diseases, Ninth Revision, Clinical Modification
ICD-10-AM: International Classification of Diseases, Tenth Revision, Australian Modification
LOS: length-of-stay
NUH: National University Hospital
OR: odds ratio
PASS: Patient Affordability Simulation System
PD: primary diagnosis
PPS: Polypharmacy Score
RSR: relative subsidy received
SD: secondary diagnosis
SES: socioeconomic status
SOC: specialist outpatient clinic
