# Supplementary material for: Processing of Electronic Medical Records for Health Services Research in an Academic Medical Center: Methods and Validation
Source: JMIR Med Inform. 2018 Dec 21;6(4):e10933. doi: 10.2196/10933 (PMC6320424; doi:10.2196/10933)
Supplement: Multimedia Appendix 1 [file medinform_v6i4e10933_app1.pdf]

**Multimedia Appendix 1.** Basic row elements and column variables in each table in Patient Affordability Simulation System (PASS).

| **Table** | **Basic row element** |
| --- | --- |
| Demographic | • Each row indicates a patient<br>• The main variables are Gender, Nationality, Race, Postal Code, Birth and Inpatient Death Date |
| Movement | • Each row indicates an outpatient visit, or a transfer record between wards and departments during inpatient visits<br>• Visit Type, Movement Type, Movement Category, Nursing/Treatment Operating Unit (OU) and Movement Time are the main variables |
| Billing | • Each row indicates a record of chargeable event or services consumed<br>• The main variables include Service Description (including descriptions of lab and radiological investigations), Service Code, Service Group, Service Quantity, Billing Time and Billing Components (e.g. Hospital Charges, Payable Amount, Subsidy Amount, Insurance Amount and Medisave Amount) |
| Pharmacy | • Each row indicates a record of dispensed drug prescription<br>• Drug Name, Drug Dosage and Drug Duration are the main variables |
| Diagnosis | • Each row indicates a record of diagnosis<br>• Diagnosis Code (ICD code), Source Department and Diagnosis Date are the main variables |
